# Early de-escalation of antibiotic therapy in hospitalized cellular therapy adult patients with febrile neutropenia

**DOI:** 10.46989/001c.94105

**Published:** 2024-02-22

**Authors:** Mariana Lucena, Kelly J Gaffney, Theresa Urban, Catherine Forbes, Pavithra Srinivas, Navneet S Majhail, Eric Cober, Sherif B Mossad, Lisa Rybicki, Betty K Hamilton

**Affiliations:** 1 Incyte (United States); 2 Medical University of South Carolina; 3 Cleveland Clinic; 4 Roswell Park Cancer Institute; 5 Janssen (United States); 6 Sarah Cannon

**Keywords:** Antibiotic de-escalation, Febrile neutropenia, Antimicrobial stewardship, Hematopoietic stem cell transplant, Chimeric antigen receptor T-cell therapy

## Abstract

Febrile neutropenia (FN) is an oncologic emergency frequently encountered in hematopoietic cell transplant (HCT) and chimeric antigen receptor (CAR) T-cell therapy patients, which requires immediate initiation of broad-spectrum antibiotics. Data regarding antibiotic de-escalation (DE) in neutropenic patients are limited, and guideline recommendations vary. A clinical protocol for antibiotic DE of broad-spectrum agents was implemented if patients were afebrile after 72 hours and had no clinical evidence of infection. The primary endpoint was the difference in the number of antibiotic therapy days between the pre-and post-DE protocol implementation group. Secondary endpoints included rates of subsequent bacteremia during index hospitalization, 30-day mortality, and hospital length of stay. Retrospective chart reviews were conducted to assess outcomes for patients who received allogeneic HCT, autologous HCT, or CAR T-cell therapy under the antibiotic de-escalation protocol (post-DE) compared to those who did not (pre-DE). The pre-DE group underwent HCT/CAR T-cell from February 2018 through September 2018 (n=64), and the post-DE group from February 2019 through September 2019 (n=67). The median duration of antibiotics was significantly lower in the post-DE group (6 days; range 3-60 days) compared to the pre-DE group (8 days; range 3-31 days) (p=0.034). There were no differences in any secondary endpoints. We conclude that antibiotic DE in neutropenic HCT or CAR T-cell therapy patients treated with broad-spectrum antibiotics for at least three days who are afebrile and without documented infection appears to be a safe and effective practice. Adopting it significantly reduces the number of days of antibiotics without compromising patient outcomes.

## Key Words

- Antibiotic de-escalation
- Febrile neutropenia
- Antimicrobial stewardship
- Hematopoietic cell transplant
- Chimeric antigen receptor T-cell therapy

## 1. Introduction

Patients receiving hematopoietic cell transplant (HCT) who present with febrile neutropenia (FN) require prompt initiation of empiric broad-spectrum antibiotics with anti-pseudomonal coverage.^1,2^ If the cause of fever remains undiagnosed, the Infectious Diseases Society of America (IDSA) 2011 guidelines recommend continuation of antibiotic therapy until absolute neutrophil count (ANC) recovery, defined as ≥ 500 cell/mm^3^.^3^ The National Comprehensive Cancer Network (NCCN) guidelines expand the IDSA recommendations and consider discontinuation or de-escalation (DE) from broad spectrum to prophylactic antibiotics if patients remain neutropenic but have resolution of fevers with no identified infectious source.^4^ Early antibiotic DE is endorsed by the European Conference on Infections in Leukemia guidelines, which recommend discontinuing antibiotics after 72 hours if a neutropenic patient has been stable since presentation and has been afebrile for at least 48 hours.^5^ While there is emerging guidance to support antibiotic DE before neutropenic recovery, there is no universal consensus to implement this practice, and the supporting evidence is based on limited data, especially in a highly immunocompromised HCT and chimeric antigen (CAR) T-cell therapy patient population.

Prolonged exposure to broad-spectrum antibiotics has many risks, particularly in immunocompromised patients. These include, but are not limited to, an increased risk of allergic reactions, development of antibiotic resistance, subsequent infection with multi-drug resistant (MDR) organisms, Clostridioidesdifficile (*C. diff*) infections, and adverse alteration of the gastrointestinal microbiome. Infections with MDR organisms, such as extended-spectrum-β-lactamase (ESBL) producing *Escherichia coli* (*E. coli*) and *Klebsiella pneumoniae* (*K. pneumoniae*), are associated with increased mortality.^6–8^ The increased risk of *C. diff* infections may lead to increased *C. diff*-associated mortality.^9^ Disruption in the gastrointestinal microbiome due to prolonged antibiotic exposure in immunocompromised patients may induce bacterial translocation and increase susceptibility to infections and other long-term complications post-HCT.^10^ Therefore, limiting the duration of exposure to broad spectrum antibiotics may prove to be a key antimicrobial stewardship practice. With growing evidence and recommendations in support of broad-spectrum antibiotic DE in neutropenic patients, the Cleveland Clinic Blood and Marrow Transplant program implemented a DE protocol in which patients undergoing allogeneic HCT, autologous HCT, and CAR T-cell therapy who have been afebrile and treated with broad spectrum antibiotics for at least 72 hours could be de-escalated to standard prophylactic antibiotics regardless of ANC. The goal of this review was to evaluate the impact of early antibiotic DE on days of antibiotic therapy and relevant patient outcomes

## 2. Materials and Methods

This was a retrospective single-center review at a tertiary academic medical center. Clinical patient data were collected from an institutional database and through electronic medical record review. The antibiotic DE protocol was developed by a program of clinical pharmacists in collaboration with HCT/CAR T-cell therapy and infectious disease physicians and implemented in February 2019. Patients admitted to the hospital under the care of the Blood and Marrow Transplant Service for HCT or CAR T-cell infusion from February 2019 through September 2019 were considered the post-DE group. These patients were compared to a historical cohort of patients hospitalized and treated from February 2018 through September 2018. The transitional period from October 2018 through January 2019 was used to develop and implement the standardized clinical protocol and to educate and onboard health professionals (e.g., physicians, advanced practice providers, nurses, pharmacists, etc.). As the standard of care at our Blood and Marrow Transplant service, all autologous and allogeneic HCT and CAR T-cell therapy recipients were hospitalized for post-cellular therapy infusion care (at the start of conditioning for HCT recipients and day 0 for CAR T-cell therapy recipients).

Inclusion criteria included any patient ≥ 18 years of age hospitalized for infusion of a cellular therapy product with a documented neutropenic fever, defined as a single oral temperature ≥38.0°C in the setting of an ANC ≤500 cells/mm^3^ per institutional practices. At the time of fever, the prophylactic antibiotic agent (ciprofloxacin 500 mg orally every 12 hours for the autologous HCT or CAR-T cell recipients and sulfamethoxazole-trimethoprim 800/160 mg orally every 12 hours for allogeneic HCT recipients) was discontinued. An antipseudomonal antibiotic (piperacillin-tazobactam as the first line with cefepime, meropenem, or aztreonam as alternatives) was initiated. Vancomycin was added for suspected catheter-related infection, suspected skin or skin structure infection, radiographic evidence of pneumonia, or if there was growth of a Gram-positive organism in blood cultures. Standard workup at onset of fever included blood cultures (including from central venous catheter), urinalysis, urine culture, chest X-ray, and any other evaluation based on clinical signs and symptoms.

The DE criteria per protocol included (1) treatment with at least three days of empiric broad-spectrum antipseudomonal antibiotics, (2) afebrile for at least 72 hours, and (3) no clinical, laboratory, or radiological evidence of infection. All patients within the pre-DE group continued broad-spectrum antibiotics until ANC recovery, defined as the first day the ANC ≥ 500 cells/mm^3^. All patients within the post-DE group had their antipseudomonal antibiotic discontinued once all criteria for DE were met and the prior prophylactic antibiotic was re-initiated. Patients with proven infection via culture data, imaging, or physical exam before antibiotic DE were excluded from this review as they did not meet predefined criteria. When implementing the DE protocol, a discussion was had amongst the multidisciplinary team regarding the timeframe to DE antibiotics (i.e., patients being afebrile for 48 or 72 hours). Although there are data to support DE in patients who are afebrile for at least 48 hours, the team felt more comfortable using a 72 hour timeframe to distinguish any new fevers arising from new infections being related to DE.^5^

The primary endpoint was the difference in number of antibiotic therapy days between the pre and post DE group. ‘Antibiotic therapy days’ was defined as the total number of days that empiric broad spectrum antipseudomonal antibiotics were administered upon presentation of initial fever, excluding any prophylactic antimicrobials. For multiple antibiotics, each day a broad-spectrum antibiotic was given was counted as 1 day of therapy towards the total number of days, excluding prophylactic antimicrobials (e.g., A patient who receives piperacillin-tazobactam for 4 days and vancomycin for 3 of these 4 days, the total antibiotic therapy days of is counted as 7 days).

**Figure 1.**
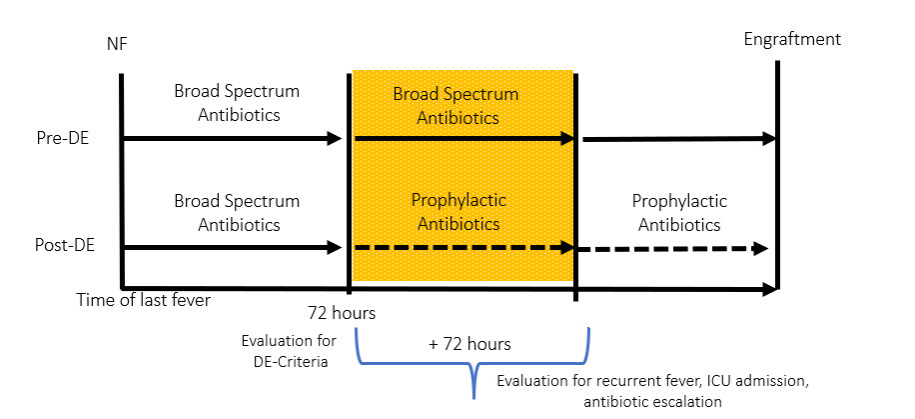
Study Design and Timeframe of Endpoints. Overview of study design to assess antibiotic de-escalation (DE). Hematopoietic cell transplant patients (HCT) and chimeric antigen receptor (CAR) T-cell therapy patients are being evaluated between the onset of febrile neutropenia (FN) and engraftment. In the pre-de-escalation (pre-DE) group, patients were started on broad-spectrum antibiotics at the onset of FN and continued until engraftment. In the post-de-escalation (post-DE) group, patients were also started on broad-spectrum antibiotics upon FN. However, these patients were eligible for antibiotic DE and switched to prophylactic antibiotics if the criteria for antibiotic DE were met. The criteria for antibiotic DE included (1) treatment with at least three days of empiric broad-spectrum antipseudomonal antibiotics, (2) afebrile for at least 72 hours, and (3) no clinical, laboratory, or radiological evidence of infection. Intensive care unit (ICU) admission, recurrent fevers, and re-escalation of antibiotic therapy were captured within a 72-hour timeframe after antibiotic DE in the post-DE group. A 72-hour timeframe was chosen to reduce the potential for additional fevers or escalation of therapy driven by new infections unrelated to DE.

Secondary endpoints included differences in rates of subsequent bacteremia during index hospitalization, 30-day mortality, hospital length of stay (LOS), and rates of *C. diff* infections. Bacteremia was defined as a laboratory-confirmed bloodstream infection (BSI). *C. diff* diagnosis was based on a polymerase chain reaction (PCR) positive result during index hospitalization. In addition, admission to the intensive care unit (ICU), recurrent fevers, and re-escalation of antibiotic therapy were evaluated within a 72-hour after antibiotic DE in the post-DE group (**Figure 1**). Re-escalation of antibiotic therapy was defined as the escalation from prophylactic to broad-spectrum antibiotic agents after DE occurred. The broad-spectrum antibacterial initiated at the time of FN was also collected.

Continuous variables were summarized as mean, standard deviation, median, minimum, and maximum and were compared between groups with the Wilcoxon rank sum test. Categorical variables were summarized as frequency counts and percentages and compared with Chi-square Fisher’s exact test.

## 3. Results

**Figure 2.**
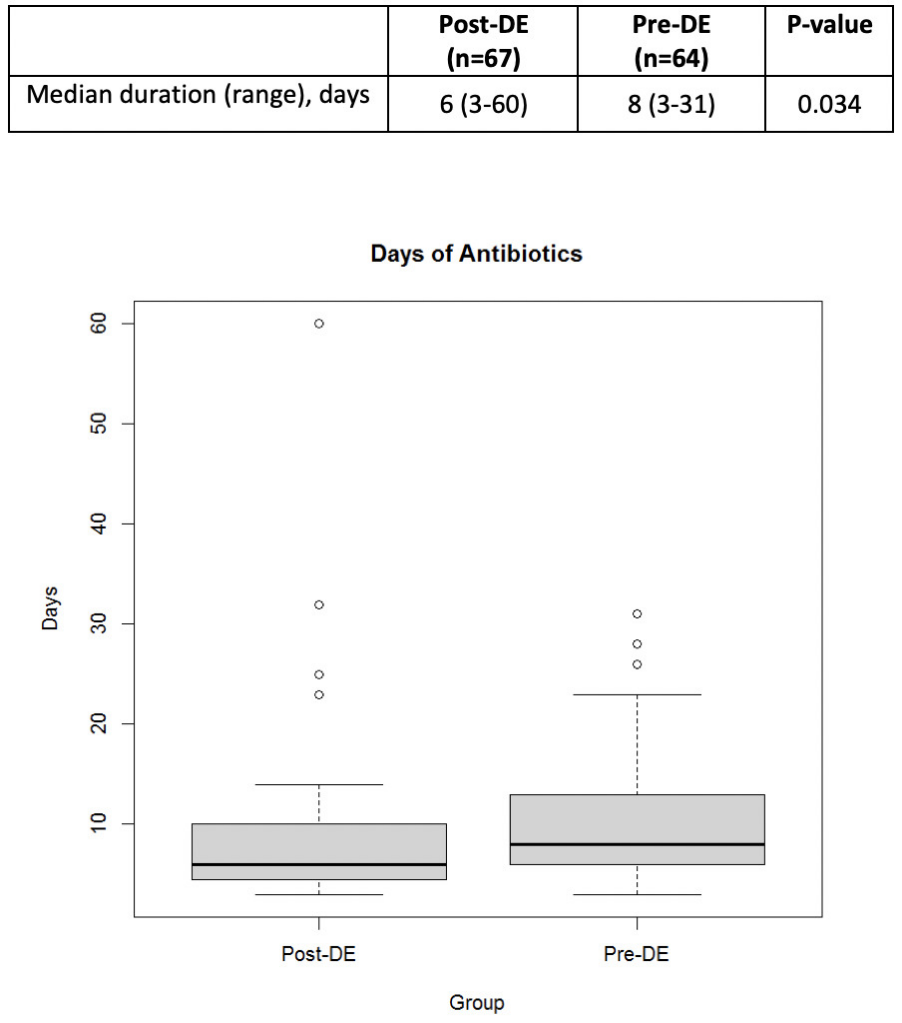
Antibiotic Days of therapy Antibiotic days of therapy in the post -de-escalation (DE) group and the pre-de-escalation (DE) group.

A total of 131 patients were included in this review. Baseline demographics were similar between groups. The median age was 59 years in the pre-DE and 60 years in the post-DE group. The most common indication for HCT was non-Hodgkin lymphoma (NHL) in both groups. A total of 41 (31.2%) patients received allogeneic HCT, 78 (59.5%) received autologous HCT, and 12 (9.2%) patients underwent CAR T-cell therapy. The most common conditioning regimen for patients undergoing autologous HCT was busulfan/cyclophosphamide/etoposide (BuCyVP) [36 (27.5%)], while those undergoing an allogeneic HCT received a reduced intensity (RIC) busulfan/fludarabine (BuFlu) [13 (9.9%)]. Given the heterogeneity of disease and cell therapy approaches, disease status at the time of HCT was significantly different between each group as there were 27 (42.2%) patients in complete response in the pre-DE compared to 23 (34.4%) in the post-DE group (P=0.05) (**Table 1**). The median total antibiotic days of therapy was significantly lower in the post-DE group compared to the pre-DE group (6 versus 8 days, respectively, p=0.034) (**Figure 2**).

**Table 1.** Patient and Transplant Characteristics

	**Pre-DE (n=64)**	**Post-DE (n=67)**	**P-value**
Median age, years (range)	59 (20-80)	60 (21-73)	0.46
Female, n (%)	29 (45.3)	34 (50.7)	0.53
Race, n (%) White Black Asian Other	55 (85.9) 3 (4.7) 2 (3.1) 4 (6.2)	62 (92.5) 4 (6) 0 (0) 1 (1.5)	0.23
Karnofsky performance status, n (%) 100 90 80 70	19 (29.7) 26 (40.6) 15 (23.4) 4 (6.2)	10 (14.9) 33 (49.3) 20 (29.9) 4 (6)	0.24
Diagnosis, n (%) NHL MM MDS AML HL MF Germ Cell ALL CML Testicular	27 (42.2) 8 (12.5) 10 (15.6) 4 (6.2) 4 (6.2) 3 (4.7) 3 (4.7) 2 (3.1) 1 (1.6) 2 (3.1)	28 (41.8) 16 (23.9) 7 (7.4) 6 (9.0) 3 (4.5) 2 (3.0) 2 (3.0) 2 (3.0) 1 (1.5) 0 (0)	0.76
Conditioning regimen, n (%) BuCyVP Melphalan BuFlu BEAM FluCy CarboVP FluCyTBI BuCy BuFluATG FluCyTBIATG FluTBI VPTBI	17 (26.6) 8 (12.5) 5 (7.8) 7 (10.9) 6 (9.4) 5 (7.8) 5 (7.8) 4 (6.2) 4 (6.2) 1 (1.6) 0 (0) 2 (3.1)	19 (28.4) 16 (23.9) 8 (11.9) 5 (7.5) 6 (9.0) 2 (3.0) 2 (3.0) 2 (3.0) 2 (3.0) 2 (3.0) 3 (4.5) 0 (0)	0.34
Transplant type, n (%) Allogeneic Autologous CAR-T	22 (34.4) 36 (56.2) 6 (9.4)	19 (28.4) 42 (62.7) 6 (9)	0.74
Disease status, n (%) Complete response Partial response No response Relapse Other	27 (42.2) 11 (17.2) 9 (14.1) 11 (17.2) 6 (9.4)	23 (34.3) 24 (35.8) 5 (7.5) 5 (7.5) 10 (14.9)	0.05

**Abbreviations:** DE, de-escalation; NHL, non-Hodgkin’s lymphoma; MM, multiple myeloma; AML, acute myeloid leukemia; MDS, myelodysplastic syndrome; HL, Hodgkin lymphoma; MF, myelofibrosis; ALL, acute lymphoblastic leukemia, CML, chronic myeloid leukemia; BuCyVP, busulfan/cyclophosphamide/etoposide; BuFlu, busulfan/fludarabine; BEAM, carmustine/etoposide/cytarabine/melphalan; FluCy, fludarabine/cyclophosphamide; CarboVP, carboplatin/etoposide; FluCyTBI, fludarabine/cyclophosphamide/total body irradiation; BuCy, busulfan/cyclophosphamide; BuFluATG, busulfan/fludarabine/anti thymocyte globulin; FluCyTBIATG, fludarabine/cyclophosphamide/total body irradiation/ anti thymocyte globulin; FluTBI, fludarabine/total body irradiation; VPTBI, etoposide/total body irradiation; CAR-T, chimeric antigen receptor T-cell

Bacteremia was identified in three (3.5%) patients in the post-DE group but none in the pre-DE group (**Table 2**). One patient experienced recurrent fever 145 hours after antibiotic DE and was re-escalated to piperacillin-tazobactam. That patient was switched to daptomycin when blood cultures grew *Rothia mucilaginosa*. The second patient experienced a fever 25 days after antibiotic DE and was re-escalated to piperacillin-tazobactam. That patient was switched to vancomycin when blood cultures grew methicillin-resistant *Staphylococcus epidermidis*. The third patient experienced recurrent fever 170 hours after DE and was re-escalated to piperacillin-tazobactam; blood cultures later grew *Pseudomonas aeruginosa*.

**Table 2.** Safety endpoints

	**Pre-DE (n=64)**	**Post-DE (n=67)**	**P-value**
Engraftment, days (± SD)*	12 (4)	13 (5)	0.30
Length of stay, days (±SD)	21 (9)	21 (9)	0.96
Recurrent fever within 72 hours of DE, n (%) Escalation of therapy, n (%)	5 (7.8) 2 (40)	5 (7.5) 5 (100)	--
30 day mortality, n (%)	0 (0)	0 (0)	--
ICU admission within 72 hours, n (%)	0 (0)	0 (0)	--
Bacteremia, n (%)	0 (0)	3 (4.5)	NS
*C. Diff* PCR positive, n (%)	0 (0)	2 (3)	0.50

**Abbreviations**: DE, de-escalation; ICU, intensive care unit; *C. diff, Clostridioides difficile*; PCR, polymerase chain reaction; SD, standard deviation; NS, not significant

*Engraftment defined as the first day ANC ≥ 500 cells/mm^3^

No patient deaths occurred within 30 days of HCT. The median LOS was 21 days for each group. *C. diff* infections were numerically higher in the post-DE group; however, this finding was insignificant (3% versus 0%, p=0.50) (**Table 2**).

In the post-DE group, there were no ICU admissions within 72 hours of DE. The rate of recurrent fever within 72 hours was 7.5% in the post-DE. All patients were restarted on broad-spectrum antibiotic therapy, which was continued through neutrophil engraftment (**Table 2**). Piperacillin-tazobactam was the most common broad-spectrum antibacterial used during FN (**Table 3**).

**Table 3.** Broad Spectrum Antibacterial Initiated Upon Febrile Neutropenia

	**Pre-DE (n=64)**	**Post-DE (n=67)**
Aztreonam, n (%)	2 (3.1)	1 (1.5)
Cefepime, n (%)	1 (1.6)	0 (0)
Gentamicin, n (%)	1 (1.6)	0 (0)
Meropenem, n (%)	4 (6.2)	0 (0)
Piperacillin-Tazobactam, n (%)	55 (85.9)	66 (98.5)

## 4. Discussion

A significant reduction in antibiotic days of therapy without notable differences in clinical outcomes was observed in this single-center retrospective review of antibiotic DE before neutrophil recovery in HCT or CAR T-cell therapy recipients. The abbreviated use of antibiotics without compromising patient safety has the potential to improve patient outcomes by limiting the emergence of MDR infections and reducing antibiotic adverse events, which are key areas of antimicrobial stewardship widely advocated by multiple organizations.^11,12^Additionally, less antibiotic use may result in decreased healthcare resource utilization and overall cost.^12–14^

Recent literature prospectively evaluated the discontinuation of antipseudomonal antibiotics in patients with hematological malignancies, regardless of neutrophil count, and found this strategy significantly decreased days of antibiotic use with no impact on mortality.^15^ A retrospective study evaluating antibiotic DE in patients who received HCT and had not engrafted reported similar results, with decreased days of antibiotics and no increase in mortality.^16^ Additionally, a retrospective analysis of antibiotic DE in HCT patients revealed no differences in mortality rates, new infections, or clinical deterioration compared to patients without early antibiotic DE. This suggests that early antibiotic DE is safe, resulting in lower healthcare costs and less exposure to broad-spectrum antibiotics.^17^ As this review concludes, the practice of early antibiotic DE has shown no negative impact on patient outcomes, including hospital LOS, morbidity, or mortality.

As previously discussed under our DE protocol, patients could be de-escalated to prophylactic antibiotics if they had received broad-spectrum antibiotics and were afebrile for at least 72 hours. The 72-hour window was considered appropriate to differentiate any additional fever or escalation of antibiotic therapy driven by new infections from being related to de-escalation. This is consistent with other clinical studies evaluating antibiotic de-escalation timeframes.^18–20^

The observed 4.5% rate of bacteremia in the post-DE group was lower than nationally reported in a similar population (10-30%).^21–24^ This discrepancy may be attributed to the study design, as those patients with a positive microbiologic diagnosis before DE were excluded.^17^ The low incidence of bacterial growth on culture beyond 72 hours supports this timeframe for DE, as there is a very low likelihood a source will later be identified.

*C. diff* infections were diagnosed via PCR only. On June 5^th^, 2018, our institution incorporated a new two-step process for diagnosing *C. diff* infection in which all PCR-positive samples underwent confirmatory toxin enzyme immunoassay (EIA) testing. Because of the timing of the implementation of this practice, the EIA data are only available for those in the post-DE; therefore, they were not used for this report. Interestingly, all *C. diff* PCR-positive samples with available EIA results were negative, suggesting the rate of *C. diff* infections may be overestimated in both cohorts.

Due to the nature of this review, there was an inherent imbalance in the comparison of groups of patients, given the evaluation of two different time points, pre- and post-antibiotic DE. Therefore, patients with neutrophil recovery in the pre-DE group were compared to neutropenic patients in the post-DE group, which we acknowledge is a potential flaw. Additionally, although the endpoints of recurrent fever and escalation of therapy were collected for both groups, these are certainly more valuable within the post-DE group, as they are strong tools to support the safety and efficacy of antibiotic DE. As such, conclusions should be primarily drawn from the data captured in the post-DE group. The rate of recurrent fever within 72 hours of antibiotic DE was low, at approximately 8% in our post-DE cohort, followed by re-escalation of therapy. Although these patients experienced recurrent fever, this did not translate into a decline in clinical status or increase in mortality, as shown by no ICU admissions within 72 hours of the antibiotic DE and no mortality events within 30 days.

Other limitations of this study include the retrospective design, which limits the ability to assess other factors that may affect outcomes. Specific pertinent clinical data were not available for the analysis of our study (i.e., oral mucositis scores, etc.). Additionally, there is inherent heterogeneity present in the groups due to the multiple disease states and cell therapy approaches, which influence several factors, including levels of immunosuppression, hospital LOS due to different admission days for conditioning or lymphodepletion regimens, and disease response status at the time of HCT/CAR T-cell. This study was predominantly descriptive, but it highlights important information to add to the literature for early DE from broad-spectrum antibiotics. More extensive prospective studies may help confirm these results and allow for further investigation of the impact of an antibiotic DE practice on subsequent infection and cellular therapy outcomes. The results of this review indicate the safety of antibiotic DE across HCT and CAR T-cell therapy patients. Despite antibiotic DE, the rates of fever resurgence and need for additional antibiotics is low, and this practice does not negatively impact subsequent infections or mortality. This DE practice has increased antimicrobial stewardship awareness amongst this interdisciplinary team, resulting in a similar antibiotic DE pilot within the malignant hematology department.

In conclusion, this review supports the safety and feasibility of antibiotic DE in patients undergoing HCT or CAR T-cell therapy who develop FN. Incorporating antibiotic DE in patients who have had at least three days of empiric broad-spectrum antimicrobials, remain afebrile for at least 72 hours, and have resolution of signs or symptoms of infection significantly decreased the days of antibiotic therapy and did not have a negative impact on outcomes.

### Note

The corresponding author was employed at the Cleveland Clinic during the conduct of this study
